# Remote Follow-up with a Mobile Application Is Equal to Traditional Outpatient Follow-up After Bariatric Surgery: the BELLA Pilot Trial

**DOI:** 10.1007/s11695-023-06587-2

**Published:** 2023-04-21

**Authors:** Cui Yang, Mia Kessler, Niki Taebi, Michael Hetjens, Christoph Reissfelder, Mirko Otto, Georgi Vassilev

**Affiliations:** 1 Department of Surgery, Medical Faculty Mannheim, University Medicine Mannheim, University of Heidelberg, Theodor-Kutzer-Ufer 1-3, 68167 Mannheim, Germany; 2 Department of Biomedical Informatics, Medical Faculty Mannheim, Heidelberg University, Mannheim, Germany

**Keywords:** Postoperative monitoring, Obesity, Mobile health app, Telemedicine, Handheld device

## Abstract

**Purpose:**

Medical follow-up after bariatric surgery is recommended. However, the compliance was poor. This study aimed to evaluate the feasibility of a smartphone-based fully remote follow-up (FU) program for patients after bariatric surgery.

**Methods:**

In the interventional group, patients were followed up using a smartphone application (app), through which questionnaires were sent regularly. Participants in the control group underwent standard FU at the outpatient clinic every three months. After 12 months, all the participants were evaluated at an outpatient clinic.

**Results:**

Between August 2020 and March 2021, 44 and 43 patients in the interventional and control groups, respectively, were included in the analysis after three patients were lost to FU, and three withdrew their informed consent because they wished for more personal contact with medical caregivers. After 12 months, total weight loss (TWL), %TWL, and percentage of excess weight loss (%EWL) did not differ between groups. There were no significant differences in the complication rates, including surgical complications, malnutrition, and micronutrition deficiency. The parameters of bioelectrical impedance analysis and quality of life did not differ between the groups. Vitamins and minerals in serum were similar in both groups except for calcium, which was significantly higher in the interventional group (2.52 mmol/L vs. 2.35 mmol/L, *p* = 0.038).

**Conclusion:**

Fully remote FU with a smartphone application is at least as effective as traditional in-person FU in an outpatient clinic after bariatric surgery. Through remote FU, patients can save time and medical professionals may have more resources for patients with more severe problems.

**Graphical Abstract:**

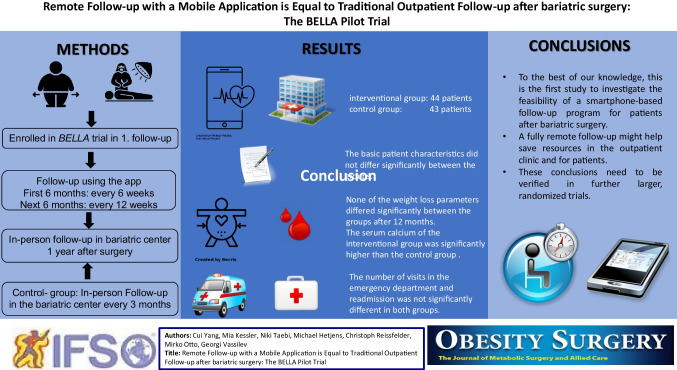

## Introduction

Over the past decades, bariatric surgery has been proven to be the most effective and durable therapy to reduce weight and comorbidities in patients with clinically severe obesity over the past decades [1–3]. Since bariatric surgeries are associated with long-term complications such as malnutrition and micronutrient deficiency [4], follow-up (FU) care after bariatric care is recommended in national and international guidelines [5–8]. Besides the early recognition of complications, loss to short-term FU (up to 3 years after surgery) is associated with insufficient weight loss (WL) [9–13], while the data for long-term FU (> three years) are controversial [14–16]. However, some remarkable problems concerning FU remain unsolved. The compliance to FU was poor: 62.1% of the patients adhered to FU after 12 months [17], and the adherence rate dropped to 44.6% during a 3-year postoperative period [18]. With the increasing number of bariatric surgeries owing to an increase in the incidence of extreme obesity, the number of patients who need postoperative FU is also rising, which results in logistical and financial challenges for bariatric centers [19].

Mobile health (mHealth), including smartphone applications (apps), has become a popular platform for delivering health services since the COVID-19 pandemic [20–24]. mHealth interventions as tools for the education and engagement of patients before bariatric surgery seem promising [25, 26]. Nevertheless, there is limited evidence regarding its effectiveness in the postoperative FU phase. In our usability study, a smartphone-based FU program was proven to be well accepted after six months [27].

This study aimed to evaluate smartphone-based health interventions for patients after bariatric surgery. We hypothesized that the mHealth intervention was at least as effective in weight loss and preventing vitamin and mineral deficiencies as a standard in-person follow-up in a bariatric center.

## Methods

### Study Design

This study was designed as a prospective, single-center analysis comparing the effects of in-person and smartphone app-based postoperative follow-up after bariatric surgery. The study was approved by the University Faculty Ethics Committee and Institutional Review Board(#2018-643N-MA) and was conducted at the university hospital. The trial is registered in the German Clinical Trials Register (DRKS00016143).

### Inclusion

Participants were patients with obesity (body mass index [BMI] ≥ 35 kg/m^2^ with one or more comorbidities [e.g., diabetes, arterial hypertension, sleep apnea] or BMI ≥ 40 kg/m^2^) who underwent primary bariatric surgery.

Participants in the interventional group were included in the BELLA trial when they presented to the outpatient clinic for their first postoperative follow-up two weeks after the surgery. Patients with re-do procedures, impaired mental state, inability to use a smartphone, language barriers, or severe postoperative complications (grade II, III, and IV according to the Clavien-Dindo classification [28]) up to the first follow-up visit were excluded.

After recruiting participants in the interventional group, the control cohort was selected based on comparable patient characteristics including median age, sex, preoperative weight, preoperative BMI, and type of surgery. All medical records were extracted from electronic patient files by the coauthors.

Participants in the control group were recruited 12 months after the bariatric surgery during the follow-up visit.

Participation for all patients was predicated on written informed consent.

### Intervention

The detailed procedures and questionnaire have been described in our previous publication [27]. Patients in the interventional group were followed up exclusively using a smartphone app instead of attending in-person visits to the outpatient clinic. The smartphone application MYONCARE™ (OnCare GmbH, Munich, Germany) was installed on their personal smartphones after obtaining informed consent. As a replacement for the in-person follow-up visits, a standardized questionnaire (see Appendix) based on the database of the German register for obesity and metabolic surgery [29] was sent to the participants via the app on scheduled appointments: every six weeks during the first nine months and then one year after the surgery. A warning message was delivered to the account of the authorized healthcare professionals if the patients' responses surpassed a predetermined threshold and suggested a potential concern (i.e., patients reported experiencing severe pain with a numerical rating scale (NRS) score > 5). Minor health problems could usually be handled by bariatric or study nurses. If patients reported major health issues, they were forwarded to physicians or the emergency department (ED) on time. The in-app communication feature of the app allows users to communicate with medical professionals. However, patients were informed not to use the in-app chat function in emergencies but to go to the ED. The app enabled the transmission of test results by general practitioners or other medical specialists. Additionally, the participants received weekly push notifications to remind them of taking vitamins or exercising. Laboratory tests were performed by primary physicians or endocrinologists six months after the surgery and then in the bariatric center 12 months after the surgery during the final visit.

### Standard Care

The control group received the usual care at the bariatric center. After the first postoperative follow-up (two weeks after surgery) with a bariatric surgeon, they were required to be present in the bariatric center for follow-up every three months in the first year after surgery. According to the German guideline [8], a postoperative follow-up visit included monitoring weight and comorbidity, assessment of eating behavior and physical activity, monitoring of vitamin and mineral supplementation, screening for mental problems and complications, such as malnutrition or malabsorption, and initiation of interventions. Laboratory tests were performed by primary physicians or in the bariatric center every six months in the first year.

### Measures

Participants in the interventional and control groups were required to attend an in-person visit to the bariatric center 12 months after surgery. Sociodemographic (sex and age) and clinical parameters (weight, height, metabolic diseases or other comorbidities) were assessed immediately before and 12 months after surgery.

#### Weight Loss Outcomes

Percentage of excess weight loss (%EWL) and percentage of excess BMI loss (%EBMIL) are the two most widely used outcome measures in bariatric surgery [30]. %EWL is less accurate as an outcome measure than absolute weight [31] and the results show significant variation by initial BMI. The percentage of total weight loss (%TWL) has been reported to be less influenced by confounding factors [32]. However, a 50%EWL is often used as a milestone for bariatric goals [32]. These weight loss parameters were calculated as follows:

$$\begin{array}{c}\%\mathrm{EBMIL}=\left[\left(\mathrm{Preoperative BMI}-\mathrm{current BMI}\right)/\left(\mathrm{preoperative BMI}-25\right)\right]\times 100\\ \%\mathrm{EWL}=\left[\left(\mathrm{Preoperative weight}-\mathrm{current weight}\right)/\left(\mathrm{preoperative weight}-\mathrm{ideal weight}\right)\right]\times 100\\ \%\mathrm{TWL}=\left[\left(\mathrm{Preoperative weight}-\mathrm{current weight}\right)/\left(\mathrm{preoperative weight}\right)\right]\times 100\end{array}$$

#### Bariatric Quality of Life Index

The Bariatric Quality of Life Index (BQL) is a validated instrument that assesses patients’ life (QoL) before and after bariatric surgery [33, 34]. The original BQL consists of 30 questions divided into two parts: non-QoL and QoL. In the German Nationwide Register, only the QoL part, consisting of 13 items with a five-point Likert scale ranging from 1 to 5 points, was included. The final score was calculated by adding all item scores, with a higher score representing a better QoL.

#### Lab Tests

The following blood tests were performed 12 months after bariatric surgery: complete blood count (CBC), liver function tests, lipid profile, iron studies, calcium, vitamin D3, vitamin B12, vitamin B1, parathyroid hormone, serum folate, and serum zinc.

#### Bioelectrical Impedance Analysis

Bioelectrical impedance analysis (BIA) is commonly performed for the evaluation of pre- and postoperative body composition, delivering the parameters of body cell mass (BCM), fat mass (FM), FM in %, and phase angle, which can be regarded as a marker of training and nutritional status [35].

### Statistical Analysis

All statistical calculations were performed with the SAS statistical program, release 9.4 (SAS Institute Inc., Cary, North Carolina, USA) and R version 3.6.3. For quantitative variables, mean and standard deviation were assessed. For qualitative factors, absolute and relative frequencies were calculated. To compare the two treatment groups with respect to baseline values, Fisher’s exact test or two-sample* t*-test was applied, as appropriate. In general, the results of a statistical test were considered statistically significant at a* p*-value of < 0.05.

## Results

### Baseline Characteristics of Participants

Between August 2020 and March 2021, of the 75 patients assessed for eligibility in the interventional group, 52 agreed to participate in the study. Eight patients dropped out of the study: one was excluded because of limited reading ability, one lost his smartphone and could not afford placement, three were lost to follow-up, and three withdrew their informed consent. Therefore, 44 participants (84.6%) were included in the final analysis. In the control group, 43 of the 74 patients assessed for eligibility were included. Medical care was provided to patients within a wide geographical area (up to 355 km). The average distance from their residence to the clinic was greater in the interventional group than the control group (42.5 km vs. 23.0 km, *p* = 0.06), but the dispersion of the data was very high (standard deviation = 62.5 in the interventional group and 25.0 in the control group).

The demographic information of the participants is presented in Table 1. The basic patient characteristics did not differ significantly between the groups.

**Table 1:** Baseline demographic characteristics of the study participants

Characteristics	Interventional group (*n* = 44)	Control group (*n* = 43)	*P* value
Age (years), mean (SD)	42.5 (10.8)	43.8 (12.4)	0.461
Sex, n (%)			0.317
Female	31 (70.4)	35 (81.4)	
Male	13 (29.6)	8 (18.6)	
Preoperative weight (kg), mean (SD)	133.8 (23.8)	136.9 (29.2)	0.581
Preoperative BMI (kg/m^2^), mean (SD)	47.0 (7.1)	48.4 (7.8)	0.416
Surgery, n(%)			0.83
RYGB	28 (63.6)	26 (60.5)	
VSG	16 (36.4)	17 (39.5)	

*RYGB*, Roux-en-Y gastric bypass; *VSG*, vertical sleeve gastrectomy; *BMI*, body mass index

### Weight Loss Outcomes

Weight was assessed during the mandatory in-person visit to the bariatric center 12 months after surgery. As shown in Table 2, none of the weight loss parameters differed significantly between the intervention and control groups.

**Table 2:** Weight loss after 12 months

Characteristics	Interventional group	Control group	*P* value
TWL	41.4 (10.1)	44.2 (17.6)	0.898
%TWL	31.2 (7.4)	32.7 (11.1)	0.459
%EWL	47.1 (10.6)	50.0 (18.1)	0.367
%EBMIL	70.3 (23.0)	71.0 (27.1)	0.898

*TWL*, total weight loss; *%EWL*, percentage of excess weight loss; *%EBMIL*, percentage of excess BMI loss; *%TWL*, percentage of total weight loss

### Complications and Readmission

One patient (2%) from each group visited the ED within 30 days, but none were readmitted. During the entire FU period, eight (18.6%) patients visited the ED in the control group: four (9.3%) patients presented with abdominal pain, one(2%) patient with nausea, one (2%) with superficial surgical site infection and one (2%) with anastomotic ulceration; only one (2%) patient was diagnosed with acute cholecystitis or symptomatic cholelithiasis and was readmitted for surgery. In the interventional group, 14 (31.8%) patients presented to the ED: seven (15.9%) with abdominal pain, one (2%) with diarrhea; six (13.6%) required readmission for surgery (five (11.4%) for acute cholecystitis or symptomatic cholelithiasis, one (2%) for bile reflux). These differences were not statistically significant (Table 3).

**Table 3:** ED visits and readmissions

Characteristics	Interventional group	Control group	*P* value
ED visit within 30 days (%)	1 (2)	1 (2)	1.000
Readmission within 30 days (%)	0 (0)	0 (0)	1.000
Overall ED visit (%)	14 (31.8)	8 (18.6)	0.218
Overall readmission (%)	6 (13.6)	1 (2)	0.110

*ED*, emergency department

### Bariatric Quality of Life Index

The BQL score was slightly higher in the intervention group, but the difference was not significant. (52.6 versus 51.9, *p* = 0.7602).

### Malnutrition and Micronutrient Deficiency

#### Albumin

The albumin level in blood did not differ significantly between the interventional and control group (39.9 mg/dL vs. 39.2 mg/dL, *p* = 0.644). In the interventional group, one of 29 patients (3.4%) had hypoalbuminemia (< 35 mg/dL), and in the control group three of 23 (13.0%) had hypoalbuminemia. The difference in hypoalbuminemia was not significant (*p* = 0.310).

#### Calcium

The serum calcium of the interventional group was significantly higher than the control group (2.52 mmol/L vs. 2.35 mmol/L, *p* = 0.038). Hypocalcemia (< 2.18 mmol/l) was diagnosed in two out of 22 patients in the control group and none in the interventional group (*p* = 0.181).

#### Lipid Profile

No significant difference in total cholesterol and triglyceride levels was found between the interventional and control group (166.4 vs. 161.9, *p* = 0.710; 94.5 vs. 105.7, *p* = 0.359, respectively). For LDL and HDL, statistical analysis was not possible owing to missing values.

#### Vitamins and Minerals

Serum levels of vitamin D, vitamin B12, and vitamin B1 were not significantly different between the groups (29.7 vs. 32.9, *p* = 0.534; 719.0 vs. 746.2, *p* = 0.854; 81.4 vs. 80.3, *p* = 0.880, respectively). There was no significant difference in the serum zinc or folate significant (14.6 vs. 16.0, *p* = 0.837; 18.0 vs. 19.5, *p* = 0.634).

Vitamin D deficiency (< 20 ng/mL) was diagnosed in eight of 27 patients (29.6%) in the interventional group and five of 17 (29.4%) in the control group (*p* = 1). No patient in either group was diagnosed with vitamin B1 or B12 deficiency.

### Compliance with Supplements of Vitamins and Minerals

Compliance with supplements was higher in the control group:69.2% of the participants in the interventional group and 88.4% in the control group stated that they had been taking vitamin and mineral supplements daily. However, this difference was not statistically significant (*p* = 0.054).

### BIA

#### BCM

BCM did not differ significantly between groups (29.4 vs. 27.8, *p* = 0.341).

#### Fat Mass and FM in %

Both FM and %FM did not significantly differ (32.8 vs. 33.2, *p* = 0.902; 34.2 vs. 35.2, *p* = 0.568).

#### Phase Angle

The phase angle was slightly higher in the intervention group (5.4 vs. 5.1°), but the difference did not reach significance (*p* = 0.086).

## Discussion

This study reports the first published German experience of follow-up after bariatric surgery using a mobile application. In our interventional cohort of patients, 84.6% were successfully followed-up with mHealth within the first 12 months. Weight loss outcomes, quality of life, malnutrition, micronutrient deficiency, and body impedance analysis results were similar to those of a cohort of patients receiving standard in-person follow-up care. These data suggest that postoperative follow-up using a mobile application is feasible in patients undergoing bariatric surgery, and a fully remote follow-up program represents a conceivable alternative to the standard in-person follow-up in an outpatient clinic.

Compliance with post-bariatric FU remains challenging and a global problem. Several patient-related risk factors for loss to FU have been identified, including younger age, persistent comorbidities, and financial challenges [9, 15]. Younger patients without comorbidities may not understand the importance of FU and may be less aware of their personal health. Therefore, they may feel it unnecessary to “waste time” visiting the hospital for a simple check-up. It is conceivable to involve this cohort in a remote FU using smartphones since 95% of adults aged 18 to 49 own a smartphone in 2021 in the USA [36], and they spend a remarkable amount of time on it. As of April 2021, the average daily time spent on a phone, excluding speaking on the phone, has climbed in recent years, totaling 4 h and 23 min [37]. Additionally, the geographical distance to the clinic/hospital may impact the readiness for follow-up.

The two most important reasons for adherence to FU after bariatric surgery are sufficient weight loss and the early detection of complications. Although the importance of FU for mid-to-long-term WL remains controversial [15, 38], a significant association between WL and FU within one year after bariatric surgery has been pointed out by several authors [9–11, 39]. In the current study, both groups had comparable BMI before surgery. After 12 months of FU using a mobile app instead of in-person visits to the bariatric center, participants in the interventional group achieved similar %EWL and %TWL compared to those with in-person visits. Some predictive factors of WL are no longer influenceable after surgery, such as sex, age, type and quality of surgery, and previous comorbid conditions. However, other factors, such as behavioral variables, can still be influenced, including physical activity and eating behaviors [40]. mHealth has been proven to be a valuable tool for managing nutrition and exercise programs in patients with overweight or obesity in diverse life situations [41–44] and plays an important role in WL in our patients. Changes in body composition after bariatric surgery have been reported in previous studies [45, 46], and the changes were similar in both groups. Moreover, the scores of bariatric quality of life were similar, which is a valid instrument for patients after bariatric surgery with better responsiveness than generic questionnaires [33].

Complication rates, including surgical complications, malnutrition, and micronutrient deficiency, were comparable between the groups. The most frequent reasons for visiting the ED were abdominal pain of unknown origin and acute cholecystitis/symptomatic cholelithiasis, which is in line with previous data in the literature [47]. The number of readmissions was slightly higher in the interventional group owing to a higher incidence of acute cholecystitis/symptomatic cholecystolithiasis; however, the difference was not statistically significant. This tendency might be explained by the fact that patients in the intervention group had easier access to medical professionals due to the chat function of the app and were thus more aware of the symptoms.

It has been well established that bariatric surgery is associated with malnutrition and micronutrient deficiency, mainly attributed to lower absorption [48]. Serum albumin level has been identified as an indicator of malnutrition, and hypoalbuminemia is usually defined as an albumin concentration of < 35 mg/dL. The serum albumin level and prevalence of hypoalbuminemia did not differ between the groups. The incidence of vitamin D deficiency after bariatric surgery has been reported to be between 10 and 73% after bariatric surgery [49]. After bariatric surgery, 3.6% of the patients were diagnosed with hypocalcemia, and the prevalence depended on the surgical type [50]. In addition to vitamin D, micronutrient deficiency was rare in both groups, and the prevalence was comparable in both groups, which might be explained by two reasons. First, all patients took part in a minimally 6-month-long multimodal concept including intensive consultation by a nutritional therapist before the surgery. Thus, they were aware of the consequences of malnutrition and micronutritional deficiencies. Second, the compliance with FU and supplement intake has proven to be the best in the first year [51]. If the patients were followed up for a more extended period with longer intervals, a dramatic drop in compliance with FU would be expected in the control group according to previous experience in the literature. Patients could benefit from a remote FU using a mobile app, since regular reminders of vitamin supplementation could be sent even years after surgery. The first indication is a significantly higher calcium level in the interventional group.

Patients could benefit from a remote FU after bariatric surgery because they do not need to travel a long distance to reach the hospital, spend time parking and waiting in a busy outpatient clinic. Furthermore, they were free to answer FU questionnaires and contact medical professionals in non-emergency anywhere and any when [27]. Medical professionals could also benefit from a remote FU: instead of organizing outpatient appointments and seeing post-bariatric patients four times per year, a standard FU could be performed, and minor problems could be solved remotely. It can take pressure away from medical professionals so that they can focus on caring for patients with more severe problems.

In our current trials, eight participants in the intervention could not be followed up using the mobile app; one patient was excluded because of limited reading ability, which had not been conscious for us during the recruitment. One patient lost his smartphone and could not afford a placement. Three patients were lost to follow-up for unknown reasons, and three withdrew their informed consent because they experienced technical problems with the app and wished for more personal contact with medical caregivers.

### Limitations

Our study had some limitations. First, selection bias could not be ruled out because it was not a randomized trial, and the control group was recruited during their 12-month follow-up appointment in the outpatient clinic. Most patients in the control group underwent bariatric surgery before the COVID-19 pandemic, while some patients in the interventional group underwent surgery during the pandemic. This might have had some impact on the willingness to participate in a remote follow-up program. Second, we only assessed data up to 12 months after surgery, which might not reflect problems in mid-to long-term follow-up. Third, owing to the characteristics of a feasibility study and missing previous data, a sample size calculation was not possible, and the number of included patients might be too low to detect differences between the groups. These conclusions need to be verified in further randomized trials. Based on the data obtained in the pilot study, we were able to perform a sample size calculation and conduct a further multicenter, randomized clinical trial to remove the ambiguities described above. In this pilot trial, we focused on the feasibility of remote follow-up and did not explicitly assess its financial aspect. To address the financial questions, the workload of healthcare providers and the travel costs of patients for in-person clinic visits will be documented and analyzed in the main trial.

## Conclusions

To the best of our knowledge, this is the first study to investigate the feasibility of a smartphone-based follow-up program for patients after bariatric surgery. Our data indicate that a fully remote follow-up program is at least as effective as a conventional in-person follow-up after bariatric surgery. Thus, a fully remote follow-up might help save resources in the outpatient clinic so that medical professionals can focus on patients with more severe problems.
